# Fourth generation e-cigarette vaping induces transient lung inflammation and gas exchange disturbances: results from two randomized clinical trials

**DOI:** 10.1152/ajplung.00492.2018

**Published:** 2019-02-06

**Authors:** Martin Chaumont, Philippe van de Borne, Alfred Bernard, Alain Van Muylem, Guillaume Deprez, Julien Ullmo, Eliza Starczewska, Rachid Briki, Quentin de Hemptinne, Wael Zaher, Nadia Debbas

**Affiliations:** ^1^Department of Cardiology, Erasme University Hospital, Université Libre de Bruxelles, Brussels, Belgium; ^2^Laboratory of Toxicology and Applied Pharmacology, Institute of Experimental and Clinical Research, Université Catholique de Louvain, Brussels, Belgium; ^3^Department of Respiratory Medicine, Erasme University Hospital, Université Libre de Bruxelles, Brussels, Belgium; ^4^Department of Clinical Chemistry, Erasme University Hospital, Université Libre de Bruxelles, Brussels, Belgium; ^5^Department of Cardiology, Centre Hospitalier Universitaire Saint-Pierre, Université Libre de Bruxelles, Brussels, Belgium

**Keywords:** arterial oxygen tension, club cell protein-16, e-cigarette, transcutaneous oxygen tension

## Abstract

When heated by an electronic cigarette, propylene glycol and glycerol produce a nicotine-carrying-aerosol. This hygroscopic/hyperosmolar aerosol can deposit deep within the lung. Whether these deposits trigger local inflammation and disturb pulmonary gas exchanges is not known. The aim of this study was to assess the acute effects of high-wattage electronic cigarette vaping with or without nicotine on lung inflammation biomarkers, transcutaneous gas tensions, and pulmonary function tests in young and healthy tobacco smokers. Acute effects of vaping without nicotine on arterial blood gas tensions were also assessed in heavy smokers suspected of coronary artery disease. Using a single-blind within-subjects study design, 25 young tobacco smokers underwent three experimental sessions in random order: sham-vaping and vaping with and without nicotine at 60 W. Twenty heavy smokers were also exposed to sham-vaping (*n* = 10) or vaping without nicotine (*n* = 10) in an open-label, randomized parallel study. In the young tobacco smokers, compared with sham-vaping: *1*) serum club cell protein-16 increased after vaping without nicotine (mean ± SE, −0.5 ± 0.2 vs. +1.1 ± 0.3 µg/l, *P* = 0.013) and vaping with nicotine (+1.2 ± 0.3 µg/l, *P* = 0.009); *2*) transcutaneous oxygen tension decreased for 60 min after vaping without nicotine (nadir, −0.3 ± 1 vs. −15.3 ± 2.3 mmHg, *P* < 0.001) and for 80-min after vaping with nicotine (nadir, −19.6 ± 2.8 mmHg, *P* < 0.001). Compared with sham vaping, vaping without nicotine decreased arterial oxygen tension for 5 min in heavy-smoking patients (+5.4 ± 3.3 vs. −5.4 ± 1.9 mmHg, *P* = 0.012). Acute vaping of propylene glycol/glycerol aerosol at high wattage with or without nicotine induces airway epithelial injury and sustained decrement in transcutaneous oxygen tension in young tobacco smokers. Intense vaping conditions also transiently impair arterial oxygen tension in heavy smokers.

## INTRODUCTION

Electronic cigarette (e-cigarette) liquid (e-liquid) is mainly constituted with propylene glycol (PG) and glycerol (GLY) in association with flavors and nicotine (15). PG is an aliphatic alcohol commonly used by industries as a humectant because of its hygroscopic properties and miscibility with water (18). GLY is an oily, hygroscopic liquid also used as a humectant (19). These molecules are “generally recognized as safe” (GRAS) for use as food additives (18, 19). When heated by an electric resistance wire (as in an e-cigarette), PG and GLY aerosolize with other substances, such as flavoring compounds and nicotine (15). This aerosol consists of a suspension mixture of gases, vapors, and aqueous particles. The latter elements condense in submicrometer to micrometer size droplets that can be inhaled into the lungs (i.e., “vaped”) (35, 52).

Oral ingestion of PG and GLY in amounts much higher than those reached by e-cigarette inhalation is not associated with significant systemic toxicity (18, 19). However, little is known about inhalation toxicity, which cannot be predicted from ingestion studies, particularly when substances induce local effects per se, as is the case with PG and GLY (11, 45, 48). As highly hygroscopic molecules, PG and GLY may dehydrate airway surface liquid (11, 18, 19, 42). This can disrupt mucociliary clearance mechanisms and lead to airway obstruction and inflammation (37, 42). In addition, PG and GLY induce hyperosmotic stress, since they do not cross biological membranes, and may induce expression and secretion of proinflammatory cytokines in the lung as observed in vitro (18, 19, 25). These inflammatory molecules can induce microvascular leakage and airway constriction by stimulating specific receptors on smooth muscle cells in the airway wall (11, 25, 31, 38). Together, these effects may also disturb mucus/surfactant rheology properties, increase surface tension, and result in small-airway collapse (47, 52). The latter phenomenon can modify the ventilation-perfusion ratio by modulating airway as well as vascular tone and therefore disturb pulmonary gas exchange (44). In addition, more than 90% of e-liquid purchased by regular users contains nicotine (9), which is released from aerosol particles deposited into the lungs, where it can induce bronchoconstriction of large airways (33).

Whether PG/GLY and/or nicotine deposition in the lungs after e-cigarette vaping triggers local inflammation or disturbs pulmonary gas exchange in humans is not known. This was assessed here in young, healthy tobacco smokers by evaluating the effects of the e-liquid vehicles PG/GLY with or without nicotine on serum and urine lung-specific proteins (i.e., pneumoproteins), serum PG, transcutaneous gas tension (an indirect measurement of arterial blood gas tension), and pulmonary function tests. We also examined the effect of pure PG/GLY vaping on arterial oxygen tension (Po_2_) in tobacco smokers suspected of coronary artery disease (CAD). Some of these results have been published previously in the form of a research letter (8).

## METHODS

### Participants and Study Design: Healthy Occasional Smokers in the First Study

On-campus paper advertisements (Erasme University Hospital, Brussels, Belgium) were used to recruit 25 healthy occasional tobacco smokers from January 2017 to November 2017. To be eligible, participants had to *1*) not smoke >20 combustible cigarettes per week, *2*) not use nicotine replacement therapy, and *3*) not use recreational drugs. Before participants entered the study, we scheduled an interview during which subjects were randomly allocated to the three experimental sessions according to a computer-generated randomization list. A medical history and physical examination were also obtained during this interview. Healthy participants were defined as follows: *1*) no acute or chronic illness, *2*) no history of cardiovascular symptoms, *3*) blood tests within the normal range, *4*) no use of medication except oral contraceptives, and *5*) no hypertension as defined by clinical guidelines (67). We also verified that volunteers were able to vape at high wattage without any symptoms of airway irritation; if participants were unable to do so, they were excluded from the study. All provided written consent after a detailed explanation of the study design, which conformed to the *Declaration of Helsinki*. The study was approved by the local Ethics Committee. The study is registered at ClinicalTrials.gov (NCT03036644).

We used a randomized, sham-controlled, single-blind, three-period, within-subjects study design in which each subject participated in each experimental exposure and served as his/her own pre/postexposure control. In random order, participants underwent the following three exposure sessions, each separated by a minimum of 1-wk washout: *1*) sham vaping, *2*) vaping without nicotine, and *3*) vaping with nicotine. A 3-h observation period followed acute exposure. While allocation and acute exposure was performed by an unblinded investigator who did not participate in any other aspect of the study, other experimenters were blinded to the study session. Participants were aware of the particular study session, since they saw the presence or absence of exhaled vapor. Twenty-one subjects performed all three experimental sessions. Two subjects finished two experimental sessions, and only one session was completed by two participants. We started data collection after 20 min of rest in a supine position in a noiseless and air-conditioned room at 23 ± 1°C. During the course of each experiment, the participant had to be silent and awake. The three study sessions for each participant began at the same time of day to avoid confounding by nychthemeral variation of cutaneous temperature and serum club cell protein-16 (CC16) concentration (1, 6). Participants were systematically screened for urine cotinine (semiquantitative detection threshold of 600 ng/ml; NarcoCheck, Paris, France) and urine tetrahydrocannabinol (semiquantitative detection threshold of 50 ng/ml, NarcoCheck), which had to be negative for inclusion. Fractional concentration of carbon monoxide (SineFuma, Breda, The Netherlands) during expiration had to be <5 ppm (17). Consumption of beverages with caffeine or alcohol, tobacco smoking, and physical exercise were not allowed for 48 h before each experimental session. A 12-h fasting state was required before each study session; participants could, however, drink water to avoid dehydration.

### Vaping Protocol in the First Study

Our pharmacy (Erasme University Hospital) mixed the e-liquid base PG/GLY (50:50 vol/vol, pharmaceutical grade; Fagron, Waregem, Belgium). Whereas one e-liquid lacked nicotine (vaping without nicotine), the other contained nicotine at a concentration of 3 mg/ml (vaping with nicotine). We used a fourth-generation e-cigarette set at 60 W (Alien 220 box mod, TFV8 baby beast tank, and a dual Kanthal coil (V8 Baby-Q2 Core; 0.4Ω dual coils; Smoke, Shenzen, China). The airflow was opened to the maximum. We followed the recommendations of the manufacturer for the preparation of the vaping devices, which were cleaned and fully filled with e-liquid before each exposure. The batteries were systematically charged to their maximum before use, and the coil was replaced after two exposures. E-cigarette exposure topography was carefully controlled by the experimenter: every 30 s, the participant inhaled vaporized aerosol for 4 s, held the aerosol for 4 s, and then exhaled. Superficial vaping was avoided by visually verifying exhalation of vapor. This procedure was repeated for 25 puffs. Sham-vaping exposure, which was strictly supervised, was identical to the active-vaping exposure but with the e-cigarette turned off. To assess the quantity of e-liquid consumed, we weighed the device before and after each exposure.

### Study Assessments in the First Study

#### Pneumoproteins, PG, and nicotine assessment.

Three blood samples were drawn as follows: just before vaping/sham-vaping exposure and 30 min and 150 min after exposure. Blood samples were immediately centrifuged at 3,500 *g* for 10 min to obtain the supernatant, which was aliquoted. Urine samples were collected 1 h before and at the end of the experimental session. Serum, plasma, and urine samples were frozen and stored at −80°C immediately after centrifugation and aliquoting.

##### cc16 and surfactant protein d.

CC16 in serum and urine was measured by latex immunoassay using a rabbit anti-CC16 antibody (Dakopatts, Glostrup, Denmark); standard CC16 was assessed at the Louvain Centre for Toxicology and Applied Pharmacology, Faculty of Medicine, Catholic University of Louvain (63). Urine retinol-binding protein (RBP) and urine and serum creatinine were quantified using the Beckman Synchron CX5 Delta Clinical System (Beckman Coulter, Fullerton, CA) (5). Concentrations of serum and urine CC16 were adjusted for serum and urine creatinine, respectively (63). The serum concentration of surfactant protein D (SPD) was determined as previously described, using a commercially available ELISA kit (Biovendor, Mokra Hora, Czech Republic) (63).

##### serum pg.

Assessment of PG was carried out using gas chromatography with a flame ionization detector (Agilent; ref. GC HP 6890-FID) after precipitation with acetonitrile (Biosolve; ref. UN1648). We used a stock solution of PG (Sigma-Aldrich; ref. 398039) at 2 mol/l in acetonitrile and an internal standard solution at 2.5 mmol/l in acetonitrile. To perform the extraction, we used 500 µl of standard, control, or sample, 100 µl of STID solution, and 900 µl of acetonitrile. These were vortexed for 3 min and then centrifuged for 10 min at 14,000 rpm at 4°C. Butylene glycol (Sigma-Aldrich no. 177652) was used as an internal standard (41).

##### serum nicotine.

Serum nicotine levels were assessed using a mass spectrometer (QQQ 6490, Agilent) with a jet stream electrospray ion source, as previously done (9).

#### Transcutaneous gas tensions and skin continuous microcirculatory blood flow.

A PeriFlux system 5000 (Perimed) explored transcutaneous oxygen (Tcpo_2_) and carbon dioxide (Tcpco_2_) tensions by means of a PF 5040 unit and a dual transcutaneous oxygen (O_2_) and carbon dioxide (CO_2_) tensions E5280 electrode. A membrane permeable to O_2_ and CO_2_ covered the E5280 electrode, which heated (44°C) the underlying tissue to maximize gas diffusion through the skin (40, 49, 65). The Tcpo_2_ was computed by a direct polarography method. The electrode consisted of a silver anode and a platinum cathode, which generate a current when the O_2_ is reduced. Thereafter, this current is converted into voltage and digitalized. Tcpo_2_ depends mainly on arterial oxygen partial pressure (Po_2_), local skin microcirculatory blood flow (SkBF), affinity of hemoglobin for O_2_, and skin metabolism (65). Since skin metabolism consumes O_2_, Tcpo_2_ is lower than Po_2_ (65). Tcpco_2_ was measured electrochemically as a result of a change in pH of an electrolyte solution located between the electrode and the membrane (40, 49). Tcpco_2_ is affected by CO_2_ partial pressure (Pco_2_), SkBF, and skin metabolism (40, 49). After the area was wiped with ethyl alcohol, a TC 550 fixation ring (Perimed) was applied to the skin on the anterior aspect of the right forearm (lower third, 5 cm distal to the antecubital fossa) at the level of the heart. Four drops of contact liquid (TC 560, Perimed) were instilled inside the TC 550 fixation ring. Avoiding superficial vessels and body hair, the electrode was placed at the same location for each of the three sessions on the basis of multiple pictures taken during the first experimental session. Calibration was performed before each session, as recommended by the manufacturer. Tcpo_2_ and Tcpco_2_ values were considered stable when variations did not exceed ±2 mmHg within 1 min. Electrode re-membraning was performed every two sessions (49). SkBF was assessed using a PeriFlow system 5000, PF 5010/5020 with the thermostatic probe 457 (Perimed) at least 5 cm from the PF 5040 Tcpo_2_/Tcpco_2_ unit. The thermostatic probe 457 emits a beam of laser light that penetrates skin tissue. By Doppler shift, there is a change in wavelength when the beam encounters a moving cell within cutaneous vessels. The probe was heated to 33°C throughout the study sessions to avoid modifications in microcirculatory blood flow induced by changes in cutaneous temperature (61).

#### Pulmonary function tests.

Nine participants were randomly selected to undergo pulmonary function tests immediately before and after exposure (sham vaping and vaping without nicotine). In this substudy, only the sham-vaping and vaping-without-nicotine experimental arms were analyzed (using the same exposure protocol as in the main experiment). Pulmonary function tests were performed with a MEC PFT body box (Medical Electronic Construction, Brussels, Belgium) before and within 5–10 min of exposure. Participants performed flow-volume curves (forced expiration and inspiration), body plethysmography (lung volumes), and measurements of the diffusion capacity of carbon monoxide in accordance with published clinical guidelines (3, 34).

### Arterial Blood Analyses and Other Cardiorespiratory Parameters in Heavy Smokers: Second Study

#### Patient recruitment and study design.

After coronary angiograms performed to exclude CAD, tobacco smokers took part in an open-label, randomized parallel study. Electronic medical records were closely scrutinized, and only patients without acute illness (e.g., acute coronary syndrome, acute heart failure, sepsis) were considered for enrolment (10). To perform the study, a coronary angiogram had to be uncomplicated (59). Patients were asked to draw an envelope containing a randomization assignment code. Twelve were randomized to sham vaping and 12 others to vaping without nicotine. Among the 12 patients allocated to vaping without nicotine, two of them were unable to complete the study due to poor tolerance of e-cigarette vaping (throat irritation, cough, or chest discomfort). One patient in the sham-vaping group withdrew informed consent before the first sham puff, and another presented with severe pain in the arm due to suspected catheter-induced radial artery vasospasm, which precluded procurement of blood gas samples. The final analysis included 10 patients in the sham-vaping arm and 10 patients in the vaping-without-nicotine arm. The study was approved by the local Ethics Committee. All patients gave written consent after a detailed explanation of the study design, which conformed to the *Declaration of Helsinki*. The study is registered at ClinicalTrials.gov (NCT03404011).

#### Vaping protocol.

The Pharmacy Department of the Erasme Hospital (Erasme University Hospital, Brussels, Belgium) prepared the vaping liquid, which consisted of pharmaceutical grade PG and GLY (50:50 vol/vol, Fagron). Flavoring was excluded to avoid confounding of outcome parameters. We did not test nicotine vaping in these settings because of the known vasoconstrictor effects of nicotine on coronary arteries. We used the same study equipment as for the main study described above. Each vaping session consisted of vaping 1 g of liquid (4-s puffs at 30-s intervals) at 60 W. The e-cigarette was weighed before exposure and after every five puffs during vaping exposure to determine the exact amount of liquid vaporized and to ensure vaping of 1 g by the end of the vaping session (mean number of puffs ± SE 17 ± 1, 4-s puffs). During the sham-vaping session, strict supervision of the participants ensured that they followed exactly the same procedure for 15 puffs with the e-cigarette turned off. Volunteers were unmasked, since they could notice that the vaping device was turned on or off.

#### Arterial blood samples.

Patients were redirected to an adjacent quiet room after their coronary angiogram to perform the study. After 20 min of comfortable rest in a sitting position, arterial samples (1.5 ml each) were drawn before exposure and 5 and 20 min after exposure (sham vaping or vaping without nicotine) via the radial arterial catheter (Radifocus Introducer II M Coat, Terumo Europe S.A.) using a heparinized syringe (safePICO, Radiometer Medical ApS). Air bubbles were removed from samples, which were gently mixed and immediately analyzed on an ABL90FLEX machine (Radiometer Medical ApS) per manufacturer recommendations. The following arterial parameters were assessed: Po_2_ and Pco_2_, pH, oximetry, electrolytes, and metabolites (69). The alveolar-arterial oxygen difference was calculated from the alveolar gas equation (60).

Throughout the experimental session, we also performed a continuous monitoring of *1*) peripheral O_2_ saturation by pulse oximetry (Spo_2_) on the index finger (27) (Intellivue MP40, Philips Belgium Commercial), *2*) heart rate, assessed with a single-derivation electrocardiogram (Intellivue MP40), *3*) Tcpo_2_ and Tcpco_2_ tensions (PeriFlow system 5000, PF 5040 with E5280 electrode, Perimed) in the anterior aspect of the arm, as recommended (49), and *4*) SkBF (61) (PeriFlow system 5000, PF 5010/5020 probe 457, Perimed). All of these measurements were performed on the opposite arm to the radial catheter.

### Data Analysis

All measurements detailed were analyzed in a blinded fashion. Details regarding missing data are provided in Supplemental Tables S1 (E.1.; first study) and S2 (E.2.; second study), which are available on https://zenodo.org/record/2553055#.XFG4P1VKipp.

#### Transcutaneous O_2_ and CO_2_ tensions in young and healthy tobacco smokers (first study).

Transcutaneous gas readings were recorded and analyzed offline (Perisoft 2.5.5, Perimed). Tcpo_2_ and Tcpco_2_ recordings began 30 min after sensor fixation and were maintained throughout the exposure session. Baseline values were obtained as the mean of 5-min recordings taken from 30 to 35 min after the sensor was fixed in place. Recording of baseline values immediately preceded vaping or sham-vaping exposure. Recordings obtained during the entire period of e-cigarette exposure were averaged to a single value. Thereafter, we followed transcutaneous gas parameters over the next 120 min, which were grouped and averaged over 12 successive 10-min intervals.

#### SkBF in young and healthy tobacco smokers (first study).

SkBF readings were recorded and analyzed offline (Perisoft 2.5.5, Perimed). SkBF recordings began 30 min after sensor fixation and were maintained throughout the session. Baseline values were obtained as the mean of 5-min recordings taken from 30 to 35 min after sensor fixation. Recording of baseline values immediately preceded vaping or sham-vaping exposure. Recordings obtained during the entire period of e-cigarette exposure were averaged to a single value. Thereafter, we followed SkBF over the next 60 min, which were grouped and averaged over six successive 10-min intervals.

#### Transcutaneous gas tension, SkBF, Spo_2_, and heart rate in heavy smokers (second study, Perimed).

Tcpo_2_ and Tcpco_2_, SkBF, Spo_2_, and heart rate recordings began 20 min after sensor fixation and were maintained throughout the session. Measurements were obtained continuously for the 5 min immediately preceding sham vaping or vaping without nicotine exposure and averaged to obtain baseline values. For this study, the 5-min and 20-min postexposure measurements consisted of the average value of continuous measurements obtained during the first 5 min after exposure and 15–20 min after exposure, respectively. Transcutaneous gas readings and SkBF were recorded and analyzed offline (Perisoft 2.5.5).

### Statistical Analysis

Continuous data were assessed for normality using the Kolmogorov-Smirnov test. If normally distributed, data were expressed as means ± SE or as the median and interquartile range [P_25_ – P_75_] if otherwise. Data are given as the difference between baseline and postexposure values (Δ). To our knowledge, this is the first research project to assess the effects of vaping on lung pneumoproteins, Tcpo_2_, and Po_2_. Therefore, sample size calculations were not possible, but a posteriori computation suggested >90% power for measurement of Tcpo_2_ and Po_2_, which was the primary outcome. A mixed-effects linear model analysis was performed with experimental sessions and time points as fixed effects and subject baselines as random effect (random intercept model). Potential autocorrelation and heteroscedasticity were included in the model. Correlation analyses used the Spearman nonparametric correlation coefficient. The R software was used; statistical significance was set at 0.05 (two-tailed tests) (47a).

## RESULTS

### Venous Blood Analyses, Transcutaneous Gas Tensions, Microcirculation, and Lung Function Tests in the First Study

The 25 participants enrolled in the first study (18 men) had a mean age of 23 ± 0.4 yr, a body mass index of 23 ± 0.4 kg/m^2^, and a median cumulative pack-year smoking history of 0.2 [0.1–0.8].

#### Pneumoproteins, PG, and nicotine assessments.

**Fig. 1.**
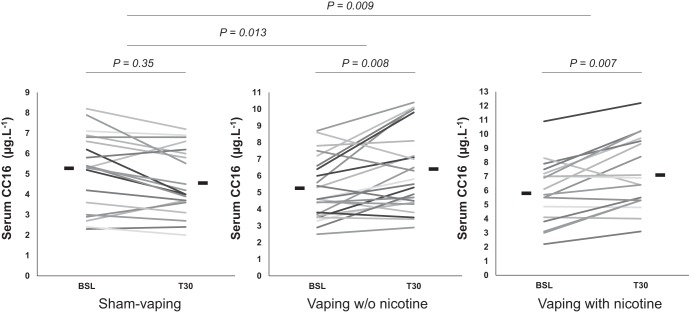
Individual changes in and overall mean value (horizontal lines) of serum club cell protein-16 (CC16) at baseline (BSL) and 30 min (T30) after sham vaping (*n* = 21), vaping without (w/o) nicotine (*n* = 24), and vaping with nicotine (*n* = 18) in the first study (healthy occasional smokers). Serum concentration of CC16 levels increased after vaping w/o and with nicotine. Horizontal brackets represent *P* values for comparison between BSL inside each experimental session (short bracket) and for comparison between sessions (long bracket). A mixed-effects linear model analysis was performed with experimental sessions and time points as fixed effects and subject baselines as random effect (random intercept model).

There were no significant differences in any biological baseline variables among the study population between the three study sessions (Table 1). Compared with sham vaping, Δ-serum CC16 increased 30 min after vaping without nicotine (−0.5 ± 0.2 vs. +1.1 ± 0.3 µg/l, *P* = 0.013; Fig. 1), and this effect persisted up to 150 min postexposure (−1 ± 0.3 vs. +1.2 ± 0.7 µg/l, *n* = 14, *P* = 0.002). Vaping with nicotine also increased Δ-serum CC16 30 min postexposure (−0.5 ± 0.2 vs. +1.2 ± 0.3 µg/l, *P* = 0.009) but not thereafter (*P* > 0.05; Table 1). Compared with sham vaping, the following parameters were not affected by vaping with or without nicotine: Δ-serum SPD; Δ-serum creatinine; Δ-urine CC16; Δ-urine creatinine; and Δ-urine RBP (all *P* > 0.05; Table 1).

**Table 1.** Concentrations of serum and urine pneumoproteins, serum propylene glycol, and nicotine according to sham vaping, vaping without nicotine, and vaping with nicotine in the first study (healthy occasional smokers)

	Sham Vaping	*P* vs. BSL	Vaping without Nicotine	*P* vs. BSL	Vaping with Nicotine	*P* vs. BSL	*P^1^*	*P^2^*	*P^3^*
Serum CC16, µg/l*									
Baseline	5.1 ± 0.4		5.2 ± 0.4		5.7 ± 0.5		0.847	0.224	0.294
T30MIN	4.6 ± 0.4	0.35	6.3 ± 0.5	0.008	7.1 ± 0.6	0.007	0.013	0.009	0.789
T150MIN	5.1 ± 0.4	0.094	7.3 ± 0.8	0.006	5.9 ± 0.7	0.436	0.002	0.078	0.127
Serum SPD, µg/l#									
Baseline	86 [66–101]		84 [66–115]		81 [62–150]		0.935	0.292	0.326
T30MIN	82 [61–104]	0.716	82 [75–124]	0.573	92 [66–145]	0.359	0.897	0.68	0.77
T150MIN	86 [69–181]	0.24	88 [72–158]	0.077	92 [75–167]	0.244	0.687	0.923	0.606
Serum creatinine, mg/dl*									
Baseline	0.89 ± 0.02		0.92 ± 0.02		0.91 ± 0.03		0.048	0.892	0.079
T30MIN	0.91 ± 0.02	0.618	0.91 ± 0.03	0.292	0.91 ± 0.03	0.31	0.275	0.684	0.145
T150MIN	0.91 ± 0.02	0.787	0.91 ± 0.04	0.27	0.88 ± 0.03	0.482	0.557	0.788	0.737
Serum CC16/serum creatinine, mg/dl*									
Baseline	5.8 ± 0.4		5.7 ± 0.4		6.3 ± 0.6		0.891	0.179	0.135
T30MIN	5.1 ± 0.4	0.424	7.1 ± 0.6	0.004	7.9 ± 0.7	0.014	0.01	0.019	0.928
T150MIN	5.7 ± 0.5	0.164	8.3 ± 1	0.002	6.7 ± 0.7	0.47	0.001	0.13	0.069
Serum PG, mmol/l#									
Baseline	0 [0–0]		0 [0–0]		0 [0–0.03]		0.72	0.988	0.732
T30MIN	0 [0–0]	0.392	0.19 [0.13–0.23]	<0.001	0.1 [0.09–0.13]	0.004	0.001	0.1	0.136
T150MIN	0 [0–0]	0.918	0.15 [0.13–0.18]	<0.001	0.1 [0.09–0.12]	0.026	0.013	0.146	0.313
Serum nicotine, ng/ml*									
Baseline					0.2 ± 0.1				
T30MIN					11.4 ± 1.5	<0.001			
T150MIN					5.4 ± 0.8	<0.001			
Urine CC16, µg/l#									
Baseline	8.8 [2.7–15.4]		11.6 [5.4–12.6]		7.2 [4.6–12.2]		0.259	0.726	0.158
End	13.2 [4.8–21.8]	0.992	17.1 [6.8–25.5]	0.622	11.2 [5.1–27.7]	0.328	0.713	0.475	0.298
Urine creatinine, g/l#									
Baseline	1.24 [0.7–2.11]		1.48 [1.02–2.09]		1.37 [0.79–1.83]		0.727	0.324	0.185
T180MIN	1.21 [0.68–1.79]	0.22	1.12 [0.57–1.57]	0.018	1.24 [0.48–1.7]	0.429	0.409	0.795	0.291
Urine CC16/urine creatinine, µg/g#									
Baseline	5.1 [2.4–14.4]		7.7 [3.8–10.8]		5.4 [2.8–10.1]		0.723	0.748	0.975
End	9.3 [6.1–16.3]	0.941	10.7 [7.6–26.2]	0.119	10.8 [4.6–21]	0.272	0.234	0.392	0.749
Urine RBP, µg/l#									
Baseline	113 [49–159]		134 [65–180]		84 [49–157]		0.183	0.269	0.17
End	103 [55–150]	0.298	79 [51–134]	0.076	113 [50–170]	0.729	0.6	0.335	0.143

*P* vs. baseline (BSL) gives *P* values for comparisons between baseline and after exposure inside each experimental arm; *P^1^* are *P* values for baseline and Δ comparisons between sham vaping and vaping without nicotine; *P^2^* are *P* values for baseline and Δ comparisons between sham vaping and vaping with nicotine; *P^3^* are *P* values for baseline and Δ comparisons between vaping without nicotine and vaping with nicotine. CC16, club cell protein-16; PG, propylene glycol; RBP, retinol-binding protein; SPD, surfactant protein-D; T30MIN, 30 min after exposure; T150MIN, 150 min after exposure; End, end of the experimental session.

* Mean ± SE;

# median [interquartile range].

**Fig. 2.**
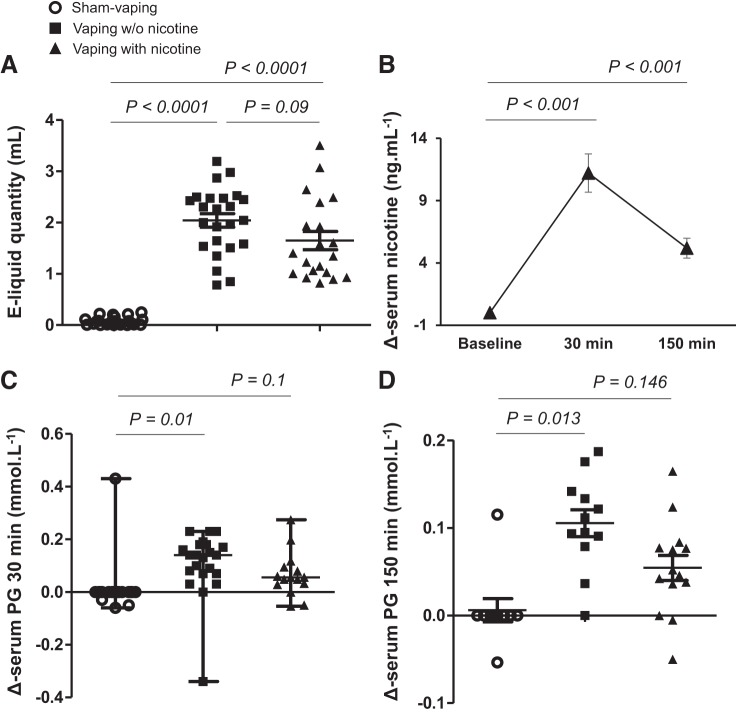
*A*: electronic cigarette liquid (e-liquid) quantity (ml) consumed after acute sham vaping (○), vaping without (w/o) nicotine (■), and vaping with nicotine (▲) in the first study (healthy occasional smokers) (means ± SE). *B*: Δ-serum nicotine 30 min and 150 min after acute vaping with nicotine (means ± SE). *C* and *D*: Δ-serum propylene glycol (PG) 30 min (median [min-max]; *C*) and 150 min (means ± SE; *D*) after sham vaping, vaping w/o nicotine, and vaping with nicotine. Horizontal brackets represent *P* values for comparison of Δ values between sessions (*A, C*, and *D*). Horizontal brackets represent *P* values for comparison between baseline inside the vaping-with-nicotine session (*B*). A mixed-effects linear model analysis was performed with experimental sessions and time points as fixed effects and subject baselines as random effect (random intercept model).

The volume of e-liquid consumed during vaping without nicotine (2.1 ± 0.2 ml) or with nicotine (1.7 ± 0.2 ml) was not significantly different (*P* = 0.09; Fig. 2*A*). Compared with sham vaping, Δ-serum PG increased after vaping without nicotine at 30 min (0 [0–0] mmol/l vs. 0.19 [0.12–0.22] mmol/l, *P* = 0.01), and this increase persisted up to 150 min postexposure (0 [0–0] mmol/l vs. 0.15 [0.13–0.18] mmol/l, *n* = 12, *P* = 0.013). Vaping with nicotine did not significantly increase Δ-serum PG 30 min (0.1 [0.03–0.13] mmol/l, *n* = 15, *P* = 0.1 vs. sham vaping) or 150 min (0.09 [0.05–0.12] mmol/l, *n* = 14, *P* = 0.146 vs. sham vaping) postexposure (Fig. 2, *C* and *D*, and Table 1).

Vaping with nicotine increased serum nicotine concentration from 0.2 ± 0.1 ng/ml to 11.4 ± 1.5 ng/ml at 30 min (*P* < 0.001 vs. baseline), which decreased to 5.4 ± 0.8 ng/ml 150 min postexposure (*P* < 0.001 vs. baseline; Fig. 2*B* and Table 1).

#### Transcutaneous gas tensions and SkBF.

**Fig. 3.**
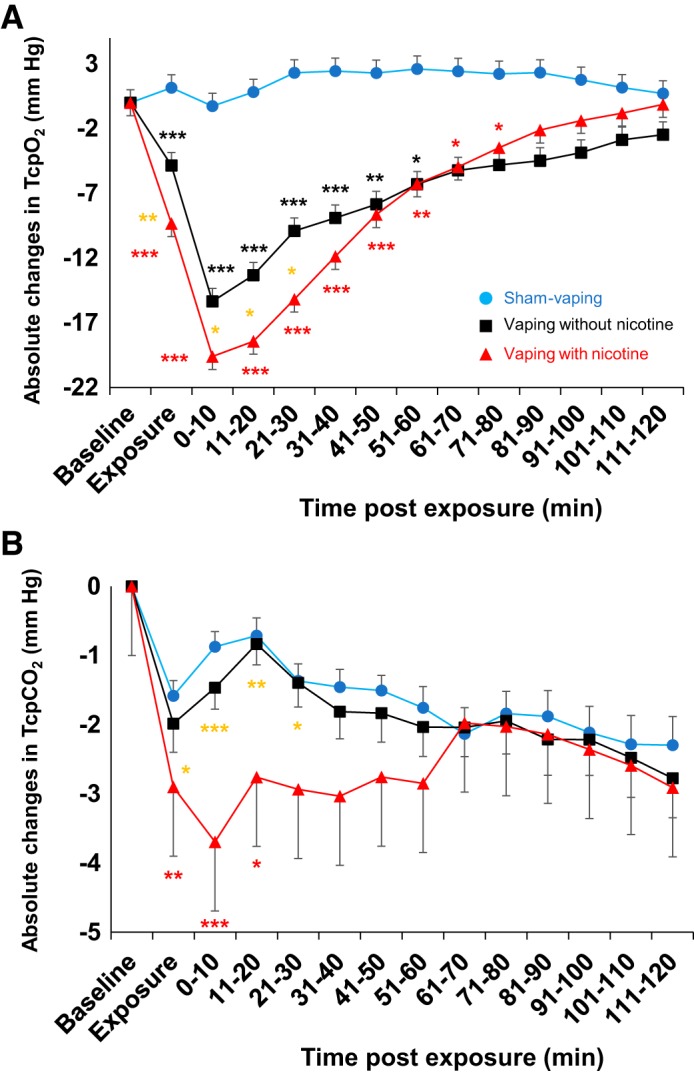
Absolute changes in transcutaneous oxygen tension (Tcpo_2_; *A*) and transcutaneous carbon dioxide tension (Tcpco_2_; *B*) over time in sham-vaping (*n* = 22, blue circles), vaping-without-nicotine (*n* = 20, black squares), and vaping-with-nicotine (*n* = 19, red triangles) sessions in the first study (healthy occasional smokers). Compared with sham vaping, vaping with or without nicotine was associated with significant decreases in Tcpo_2_ for 80 and 60 min postexposure, respectively. Compared with vaping without nicotine, vaping with nicotine decreased Tcpo_2_ for 30 min postexposure. Compared with sham vaping, vaping with nicotine was associated with significant decreases in Tcpco_2_ that persisted for 20 min postexposure. Black and red asterisks denote *P* values for comparisons with sham vaping; gold asterisks denote *P* values for comparisons between vaping without nicotine and vaping with nicotine (*P < 0.05, **P < 0.01, ***P < 0.001). Data presented are means ± SE. A mixed-effects linear model analysis was performed with experimental sessions and time points as fixed effects and subject baselines as random effect (random intercept model).

Baseline Tcpo_2_ and Tcpco_2_ did not differ significantly between sham vaping (84.9 ± 2.7 and 37.9 ± 0.9 mmHg, respectively), vaping without nicotine (84.6 ± 2.2 and 37.5 ± 1.2 mmHg, respectively), and vaping with nicotine (80.5 ± 2 and 38 ± 1.3 mmHg, respectively) (all *P* > 0.05). Compared with sham-vaping, *1*) vaping without nicotine decreased Δ-Tcpo_2_ during the 60 min after vaping, with the nadir reached during the first 10 min after exposure (–0.3 ± 1 vs. −15.3 ± 2.5 mmHg, *P* < 0.001), *2*) vaping with nicotine decreased Δ-Tcpo_2_ during the ensuing 80 min after exposure, with the nadir reached during the first 10 min (−0.3 ± 1 vs. −19.6 ± 2.8 mmHg, *P* < 0.001). Vaping without nicotine decreased Δ-Tcpo_2_ less than vaping with nicotine during the period of exposure (−4.8 ± 1.1 vs. −9.3 ± 1.8 mmHg, *P* < 0.01) and 30 min postexposure (−9.9 ± 2.3 vs. −15.2 ± 2 mmHg, *P* = 0.02; Fig. 3*A*). Δ-Tcpco_2_ decreased for 20 min in the nicotine vaping arm, with the nadir at 10 min postexposure (−0.9 ± 0.2 vs. −3.7 ± 1.1 mmHg, *P* < 0.001 vs. sham vaping). Sham vaping and vaping without nicotine did not modify Δ-Tcpco_2_ (all *P* > 0.05; Fig. 3*B*). None of the three experimental sessions modified SkBF (all *P* > 0.05).

#### Pulmonary function tests.

Compared with sham vaping, vaping without nicotine decreased Δ-FEF-25% (forced expiratory flow at 25% of pulmonary volume) (−0.03 ± 0.07 vs. −0.32 ± 0.05 l/s, *P* = 0.03) and forced expiratory volume in 1 s/forced vital capacity (0.26 ± 0.73 vs. −3.39 ± 0.55%, *P* = 0.014). All other pulmonary function tests and diffusing capacity variables were not modified by vaping or sham vaping (all *P* > 0.05; Table 2).

**Table 2.** Functional pulmonary variables (flow-volume curves, body pletysmography, and diffusion capacity of carbon monoxide) in the study population according to sham vaping or vaping without nicotine immediately before and within 5 and 10 min after exposure (n = 9) in the first study (healthy occasional smokers)

	Sham Vaping	*P* vs. BSL	Vaping without Nicotine	*P* vs. BSL	*P^1^*	*P^2^*
Forced expiratory volume in 1 s, l						
Baseline	4.5 [4–4.6]		4.4 [4.2–4.6]		0.387	
After exposure	4.2 [4–4.6]	0.592	4.3 [3.9–4.6]	0.021		0.187
Forced expiratory volume in 1 s/forced vital capacity, %						
Baseline	82.2 [77.5–84.1]		83.5 [76.3–85.7]		0.152	
After exposure	82 [77.7–84.8]	0.79	81 [74–82.6]	0.002		0.014
Peak expiratory flow, l/s						
Baseline	7.8 [7.4–9.8]		8.5 [7.2–9.3]		0.551	
After exposure	9.2 [7.4–9.9]	0.538	7.85 [7–9.8]	0.633		0.441
Forced expiratory flow-75%, l/s						
Baseline	6.9 [6.1–8.6]		7.2 [6.1–8.8]		0.808	
After exposure	7.1 [5.5–8.8]	0.522	6.9 [5.9–8.2]	0.112		0.486
Forced expiratory flow-50%, l/s						
Baseline	5 [3.6–5.4]		4.8 [4–6.1]		0.285	
After exposure	4.8 [3.6–5.1]	0.588	4.2 [3.7–5.5]	0.009		0.124
Forced expiratory flow-25%, l/s						
Baseline	2.2 [1.5–2.5]		2.5 [1.7–2.6]		0.321	
After exposure	2.1 [1.6–2.5]	0.764	2 [1.4–2.3]	0.002		0.033
Forced midexpiratory flow rate, l/s						
Baseline	4.5 [3.1–4.7]		4.2 [3.5–5.4]		0.272	
After exposure	4.2 [3.1–4.6]	0.545	3.7 [3.1–4.9]	0.003		0.071
Airway total resistance, cmH_2_O·l^−1^·s^−1^						
Baseline	3.75 [3.2–5]		4 [3.35–4.5]		0.98	
After exposure	3.9 [3.4–4.5]	0.661	4.5 [3.8–5.9]	0.089		0.13
Intrathoracic gas volume, l						
Baseline	3.2 [2.9–4]		3.5 [2.7–4]		0.922	
After exposure	3.5 [3–3.8]	0.943	3.1 [2.7–3.7]	0.486		0.657
Total lung capacity, l						
Baseline	6.9 [6.2–8]		6.7 [6.2–7.9]		0.635	
After exposure	7.2 [6.7–7.8]	0.649	6.6 [5.9–7.7]	0.517		0.437
Residual volume, l						
Baseline	1.5 [1.1–2.4]		1.4 [1.2–2.5]		0.904	
After exposure	1.8 [1.6–2.25]	0.57	1.5 [1.2–2.2]	0.59		0.435
Residual volume/total lung capacity, %						
Baseline	26 [19–30]		21 [19.5–31]		0.47	
After exposure	27 [23.5–29.5]	0.452	23 [19.5–28]	0.657		1
Diffusion capacity of carbon monoxide, ml·min^−1^·mmHg^−1^						
Baseline	32.65 [28.4–38.3]		34.1 [23.4–41]		0.712	
After exposure	32.1 [26.1–37.7]	0.401	30.7 [26.6–43.1]	0.398		0.237

Values are medians [interquartile ranges]. *P* vs. baseline (BSL) gives *P* values for comparisons between baseline and after exposure; *P^1^* are *P* values for baseline comparisons between sham vaping and vaping without nicotine; *P^2^* are *P* values for Δ comparison between sham vaping and vaping without nicotine.

#### Correlation between variables.

**Fig. 4.**
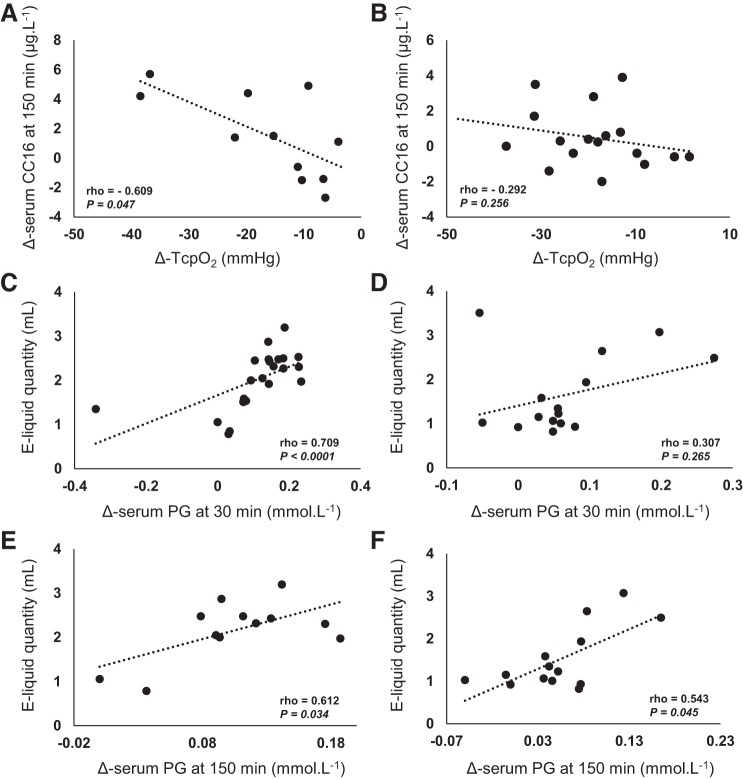
Correlation of Δ-serum club cell protein-16 (CC16) 150 min after acute vaping without nicotine (*A*), vaping with nicotine (*B*), and nadir of Δ-transcutaneous oxygen tension (Tcpo_2_) in the first study (healthy occasional smokers). Positive correlation of electronic cigarette liquid (e-liquid) quantity consumed and Δ-serum propylene glycol (PG) 30 and 150 min after vaping without nicotine (*C* and *E*, respectively) and vaping with nicotine (*D* and *F*, respectively). Correlation analyses used the Spearman nonparametric correlation coefficient.

A negative correlation was found in the vaping-without-nicotine arm between Δ-Tcpo_2_ 10 min after exposure and Δ-serum CC16 150 min after exposure (Spearman’s ρ = −0.609, *n* = 11, *P* = 0.047*;* Fig. 4*A*). The latter correlation was not observed in the vaping-with-nicotine arm (Spearman’s ρ = −0.292, *n* = 17, *P* = 0.256; Fig. 4*B*).

A positive correlation was found between the quantity (ml) of e-liquid vaped during the nicotine-free vaping session and Δ-serum PG 30 min (Spearman’s ρ = 0.709, *n* = 22, *P* < 0.0001*;* Fig. 4*C*) and Δ-serum PG 150 min (Spearman’s ρ = 0.612, *n* = 12, *P* = 0.034*;* Fig. 4*E*). In the case of nicotine vaping, a correlation was found only between the quantity of e-liquid vaped and Δ-serum PG 150 min after exposure (Spearman’s ρ = 0.543, *n* = 14, *P* = 0.045; Fig. 4*F*).

**Fig. 5.**
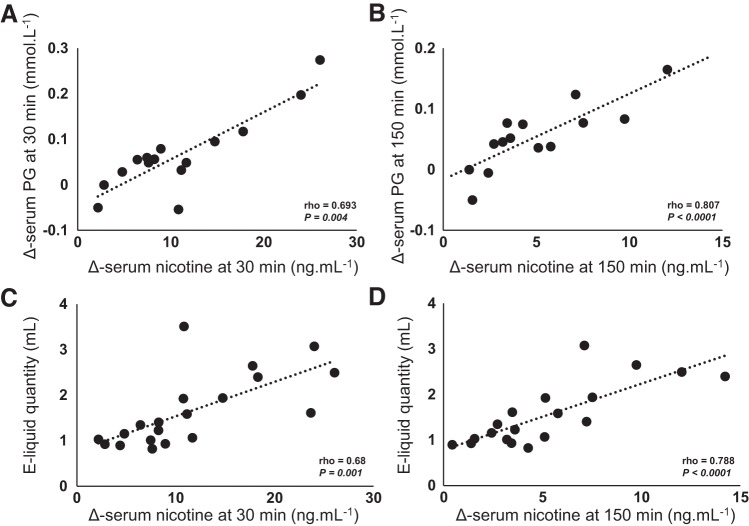
Positive correlation of Δ-serum propylene glycol (PG) and Δ-serum nicotine 30 min (*A*) and 150 min (*B*) after acute vaping with nicotine exposure in the first study (healthy occasional smokers). Positive correlation of electronic cigarette liquid (e-liquid) quantity (ml) consumed and Δ-serum nicotine 30 min (*C*) and 150 min (*D*) after acute vaping with nicotine exposure. Correlation analyses used the Spearman nonparametric correlation coefficient.

Finally, in the nicotine vaping arm, strong positive correlations were found between Δ-serum PG 30 min and Δ-serum nicotine 30 min (Spearman’s ρ = 0.693, *n* = 15, *P* = 0.004; Fig. 5*A*) after exposure; between Δ-serum PG 150 min and Δ-serum nicotine 150 min (Spearman’s ρ = 0.807, *n* = 14, *P* < 0.0001; Fig. 5*B*); between the quantity of e-liquid vaped and Δ-serum nicotine 30 min (Spearman’s ρ = 0.68, *n* = 20, *P* = 0.001; Fig. 5*C*) and Δ-serum nicotine 150 min (Spearman’s ρ = 0.788, *n* = 19, *P* < 0.0001; Fig. 5*D*) after acute exposure.

### Arterial Blood Analyses, Pulse Oximetry, Heart Rate, Transcutaneous Gas Tension, and Microcirculation in the Second Study

The mean age of the 20 patients (17 men) who completed the study was 54 ± 2 yr with a body mass index of 29 ± 1 kg/m^2^ and a cumulative smoking history of 34 ± 3 pack-years. Established CAD was documented in 65% (*n* = 13) patients. Thirty percent (*n* = 6) of the patients had concurrent chronic obstructive pulmonary disease (COPD). There were no notable differences in either preexisting medical conditions (Table 3) or in baseline variables between the sham-vaping and vaping groups (Table 4).

**Table 3.** Medical features of the study population according to sham vaping and vaping without nicotine arms in the second study (heavy smokers)

Variables	All Participants	Sham Vaping	Vaping without Nicotine	*P*
*N*	20	10	10	
Age, yr*	54 ± 2	55 ± 3	53 ± 3	0.719
Men, %	85	80	90	1
BMI, kg/m^2^*	29 ± 1	31 ± 2	26 ± 2	0.086
Cigarettes, pack-year*	34 ± 3	34 ± 4	34 ± 4	1
Comorbidities				
Hypertension, %	80	80	80	1
Diabetes, %	15	10	20	1
Established CAD, %	65	60	70	1
LVEF <50%, %	60	70	50	0.648
Dyslipidemia, %	50	50	50	1
History of AMI, %	15	10	20	1
Established COPD, %	30	30	30	1
Medications				
RAS blockade, %	60	60	60	1
β-Blockers, %	50	60	40	0.655
Diuretics, %	25	30	20	1
Antiplatelets agents, %	60	70	50	0.648
Anticoagulent agents, %	10	10	10	1
Calcium channel blockers, %	25	30	20	1
Statins, %	30	30	30	1

BMI, body mass index; CAD, coronary artery disease; LVEF, left ventricular ejection fraction; AMI, acute myocardial infarction; COPD, chronic obstructive pulmonary disease; RAS, renin-angiotensin system.

* Data presented are means ± SE.

#### Arterial blood sample analyses.

**Fig. 6.**
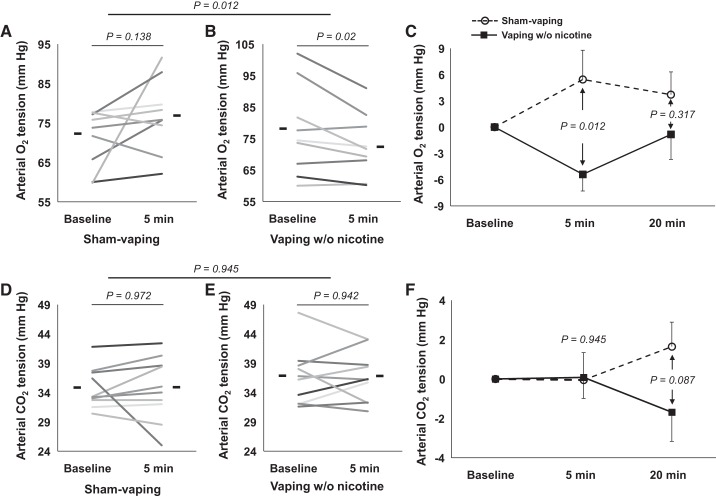
Individual changes in and overall mean values (horizontal lines) of arterial O_2_ and CO_2_ tensions at baseline and 5 min after sham vaping (*n* = 10; *A* and *D*, respectively) and vaping without nicotine (*n* = 10; *B* and *E*, respectively) in the second study (heavy smokers). Results of statistical tests are also provided (horizontal brackets). Absolute change in arterial O_2_ and CO_2_ tensions at baseline, 5 min, and 20 min after sham vaping (dashed line, ○; *C* and *F*), and vaping without nicotine (solid line, ■; *C* and *F*). Results of statistical tests comparing sham vaping and vaping without nicotine are also provided. A mixed-effects linear model analysis was performed with experimental sessions and time points as fixed effects and subject baselines as random effect (random intercept model). Data are means ± SE.

Compared with sham vaping, 5 min after the exposure, vaping decreased: *1*) Δ*-*Po_2_ (+5.4 ± 3.3 vs. −5.4 ± 1. 9 mmHg, respectively, *P =* 0.012; Fig. 6, *A*–*C*) (difference between groups; 10.8 [95% CI, 8.4–13.2] mmHg); *2*) Δ-So_2_ (+0.9 ± 0.6 vs. −0.8 ± 0.3%, respectively, *P =* 0.023; and *3*) Δ-oxyhemoglobin fraction (+1 ± 0.5 vs. −0.6 ± 0.4%, respectively, *P* = 0.028). Compared with sham vaping, 20 min after the exposure, vaping decreased: Δ-HCO_3_^−^ (+1 ± 0.8 vs. −1.1 ± 0.6 mmol/l, respectively, *P* = 0.045) and Δ-base excess (+1.3 ± 1 vs. −1.6 ± 0.8 mmol/l, respectively, *P* = 0.042). Pco_2_ (Fig. 6, *D*–*F*), electrolytes, and metabolites were not modified by vaping or sham vaping (all *P* > 0.05; Table 4).

**Table 4.** Blood gases, oxymetry, electrolyte, and metabolite analyses in arterial blood samples, transcutaneous gases tensions, and finger pulse oxymetry before and 5 and 20 min after exposure in sham-vaping and vaping arms in the second study (heavy smokers)

	Sham-vaping			Vaping					
	BSL	T5MIN^1^	T20MIN^2^	BSL	T5MIN^3^	T20MIN^4^	*P* BSL*	*P* Δ1 vs. Δ3^#^	*P* Δ2 vs. Δ4^†^
Arterial blood gas									
Ph	7.43 ± 0.01	7.44 ± 0.01	7.43 ± 0.01	7.43 ± 0.01	7.42 ± 0.01	7.42 ± 0.01	0.798	0.123	0.229
Pco_2_, mmHg	34.7 ± 1.1	34.7 ± 1.7	36.1 ± 1.8	36.6 ± 1.5	36.7 ± 1.3	34.6 ± 2.2	0.419	0.945	0.087
Po_2_, mmHg	72 ± 2.6	77.4 ± 2.8	76.3 ± 3.9	78.1 ± 4.3	72.7 ± 3	78.5 ± 3.5	0.207	0.012	0.317
HCO_3_^−^, mmol/l	23.8 ± 0.7	24 ± 0.9	24.5 ± 0.8	24.5 ± 0.7	24 ± 0.6	23.1 ± 1.1	0.531	0.467	0.045
O_2_ alveolar-arterial gradient	34.4 ± 2.1	29 ± 2.5	28.3 ± 2.1	29.8 ± 4.7	34.6 ± 4	31.9 ± 4.3	0.349	0.015	0.052
Base excess, mmol/l	−1.01 ± 0.88	−0.83 ± 1.23	−0.07 ± 1.08	−0.03 ± 0.92	−0.68 ± 0.79	−1.99 ± 1.56	0.516	0.526	0.042
Oxymetry									
Hematocrit, %	41.7 ± 1.7	42.1 ± 1.7	44.1 ± 1.4	41.8 ± 0.9	42.3 ± 1	41 ± 2	0.961	0.946	0.213
Hemoglobin, g/dl	13.6 ± 0.6	13.7 ± 0.5	14.4 ± 0.4	13.6 ± 0.3	13.8 ± 0.3	13.4 ± 0.7	0.976	0.936	0.207
So_2_, mmHg	95.2 ± 0.6	96.1 ± 0.6	96 ± 0.6	95.8 ± 0.8	95 ± 0.8	96.1 ± 0.6	0.527	0.023	0.625
Oxyhemoglobin fraction, %	92.7 ± 0.7	93.7 ± 0.4	93.4 ± 0.7	93.2 ± 0.9	92.6 ± 0.8	93.6 ± 0.7	0.577	0.028	0.721
O_2_ alveolar-arterial gradient, mmHg	34.4 ± 2.1	29 ± 2.5	28.3 ± 2.1	29.8 ± 4.7	34.6 ± 4	31.9 ± 4.3	0.349	0.015	0.052
Carboxyhemoglobin fraction, %	1.91 ± 0.32	1.77 ± 0.35	1.97 ± 0.33	1.96 ± 0.42	1.88 ± 0.38	1.93 ± 0.41	0.921	0.54	0.562
Methemoglobin fraction, %	0.76 ± 0.06	0.7 ± 0.06	0.68 ± 0.05	0.72 ± 0.08	0.67 ± 0.08	0.72 ± 0.08	0.67	0.878	0.281
Electrolytes									
Sodium, mmol/l	139.5 ± 0.9	140 ± 0.8	139.2 ± 0.8	139.8 ± 0.6	140.2 ± 0.5	140.9 ± 0.8	0.768	0.909	0.171
Potassium, mmol/l	3.5 ± 0.2	3.5 ± 0.2	3.7 ± 0.1	3.8 ± 0.1	3.8 ± 0.1	3.6 ± 0.2	0.126	0.76	0.094
Chlore, mmol/l	109.8 ± 1.6	109.5 ± 1.8	107.9 ± 1	108.3 ± 1.1	108.9 ± 1	111.5 ± 2.5	0.484	0.74	0.068
Ionized calcium, mmol/l	1.103 ± 0.04	1.108 ± 0.04	1.143 ± 0.031	1.158 ± 0.016	1.15 ± 0.012	1.112 ± 0.056	0.275	0.803	0.096
Metabolites									
Glucose, mg/dl	106 ± 11	105 ± 11	110 ± 10	105 ± 7	99 ± 3	94 ± 6	0.933	0.476	0.164
Lactate, mmol/l	0.69 ± 0.11	0.76 ± 0.12	0.71 ± 0.11	0.64 ± 0.08	0.69 ± 0.06	0.57 ± 0.07	0.741	0.768	0.166
Cutaneous microcirculation									
Tcpo_2_,%	61.8 ± 3.9	67.8 ± 6.4	65.1 ± 5	64.7 ± 3.6	63.5 ± 4.7	62.8 ± 5	0.684	0.041	0.125
Tcpco_2_, %	34.6 ± 1.1	33.9 ± 1.7	34.7 ± 1.5	35.1 ± 1.7	37.9 ± 2.1	39 ± 2.2	0.853	0.033	0.02
SkBF, PU	21 ± 9	17 ± 6	19 ± 7	16 ± 3	19 ± 4	15 ± 2	0.474	0.017	0.618
Pulse oximetry, %	96.1 ± 0.4	97.4 ± 0.5	96.7 ± 0.5	97.1 ± 0.7	95.8 ± 0.6	96.4 ± 0.7	0.231	<0.0001	0.036
Heart rate, beats/min	82 ± 3	79 ± 2	77 ± 2	77 ± 6	87 ± 9	84 ± 8	0.769	0.002	0.005

Values are means ± SE. BSL, baseline; T5MIN,  5 min postexposure; T20MIN , 20 min postexposure; Ph, power of hydrogen; Pco_2_, arterial carbon dioxide tension; Po_2_, arterial oxygen tension; HCO_3_^−^, bicarbonate anion; So_2_, arterial oxygen saturation; Tcpo_2_,  transcutaneous oxygen tension; Tcpco_2_, transcutaneous carbon dioxide tension; SkBF, skin microcirculatory blood f low.

* *P* values for comparisons between baselines in sham-vaping and vaping arms;

# *P* values for baseline differences at 5-min (Δ) comparisons between sham-vaping and vaping arms;

† *P* values for baseline differences at 20-min (Δ) comparisons between sham-vaping and vaping arms.

#### Pulse oximetry, heart rate, transcutaneous gas tension, and skin microcirculation.

**Fig. 7.**
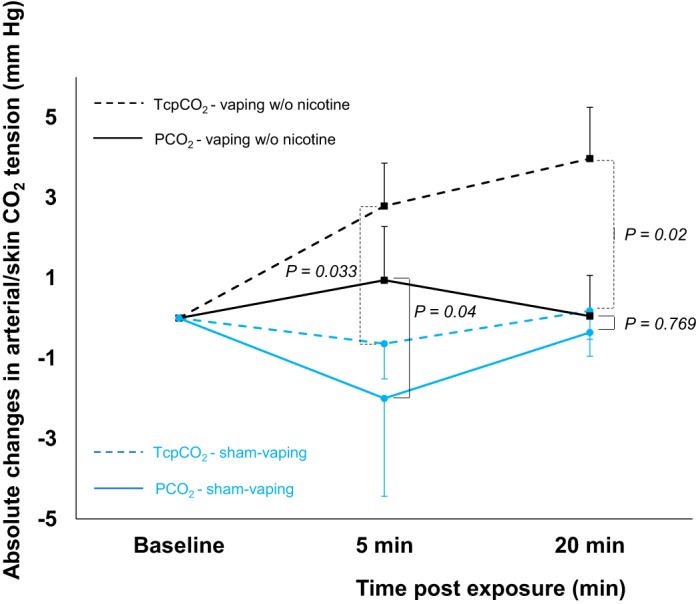
Absolute changes in transcutaneous oxygen tension (Tcpco_2_, dashed line) and arterial CO_2_ partial pressure (Pco_2_, solid line) at baseline and 5 and 20 min after acute sham vaping (blue line, *n* = 5) and vaping without (w/o) nicotine (black line, *n* = 7). A mixed-effects linear model analysis was performed with experimental sessions and time points as fixed effects and subject baselines as random effect (random intercept model). Data presented are means ± SE.

Compared with sham vaping, 5 min after vaping exposure, vaping *1*) decreased Δ*-*Spo_2_ (+1.3 ± 0.4 vs. −1.3 ± 0.5%, respectively, *n* = 14, *P* < 0.0001); *2*) increased Δ-heart rate (−2.3 ± 2.1 vs. +10.6 ± 4.3 beats/min, respectively, *n* = 14, *P =* 0.002); *3*) decreased Δ*-*Tcpo_2_ (+6 ± 3.4 vs. −1.2 ± 2.1 mmHg, respectively, *n* = 12, *P* = 0.041); *4*) increased Δ*-*Tcpco_2_ (−0.6 ± 0.9 vs.+2.8 ± 1.1 mmHg, respectively, *n* = 12, *P* = 0.033*;* Fig. 7); and *5*) increased Δ-SkBF (−4.4 ± 3.4 vs. +3.4 ± 1.8 mmHg, respectively, *n* = 12, *P* = 0.017). Compared with sham vaping, effects of vaping were still present 20 min after exposure for Δ*-*Spo_2_ (+0.7 ± 0.6 vs. −0.7 ± 0.5%, respectively, *n* = 14, *P* = 0.036), for Δ*-*Tcpco_2_ (+0.2 ± 0.7 vs. +4 ± 1.3 mmHg, respectively, *n* = 12, *P* = 0.02) and for Δ-heart rate (−3.9 ± 1.9 vs. +7.3 ± 3.3 beats/min, respectively, *n* = 14, *P* = 0.005; Table 4).

## DISCUSSION

E-cigarette inhalation of pharmaceutical grade, nicotine-free PG/GLY mix (50:50 vol/vol) in young and healthy occasional tobacco smokers induced irritation of the lower respiratory tract as reflected by increases in serum CC16 levels and evidence of small airway constriction in pulmonary function tests. The latter effects were accompanied by sustained decrements in Tcpo_2_. Furthermore, vaping the PG/GLY mixture decreased Po_2_, oxyhemoglobin fraction, and Tcpo_2_ in heavy smokers. These results strongly suggest that vaping disturbs pulmonary gas exchange. Surprisingly, similar variations were observed when the PG/GLY mix with nicotine was vaped, suggesting that nicotine is not responsible for the observed effects.

### Serum PG and Vaping

Deposits of PG/GLY after e-cigarette inhalation localize primarily on the epithelium of large airways but also reach the terminal bronchioles and alveoli (35). The airway epithelium separates the air-filled compartment of the respiratory system from the vascular compartment and regulates solute and water exchange between these two compartments (28). To our knowledge, the fate of e-cigarette PG aerosol deposits in airways and their interactions with epithelial cells has not yet been studied in humans. In our healthy participants, serum PG acutely increased after vaping without nicotine and persisted up to 150 min after exposure. Fortunately, the serum PG concentrations achieved are unlikely to cause any systemic toxicity (18). The elimination half-life of PG is ~4 h, as it is quickly metabolized into lactic acid by alcohol dehydrogenase in the liver and then to pyruvic acid, which enters the citric acid cycle and is further metabolized into CO_2_ and water (18). Our findings support experimental data from rodent studies that PG transits swiftly from the lung to the bloodstream and does not accumulate significantly in the plasma, even after repeated daily inhalation (45). In the latter study, however, some evidence of PG accumulation within the lung was found. This is presumably because PG exposure induces deep airway inflammation that may cause slight retention of PG in the lungs (45). Indeed, beside regulation of water-solute exchange, the pulmonary epithelium also removes airborne particulate matter from the airways and orchestrates the immune response (28, 68). Interaction of PG/GLY and nicotine aerosols with airway epithelium may trigger pulmonary clearance mechanisms, including recruitment of inflammatory cells, with subsequent lung inflammation and PG retention (28, 35, 68).

### Pneumoproteins After Vaping

We found that serum levels of the anti-inflammatory protein CC16 increased acutely after vaping PG/GLY with and without nicotine. This small protein is expressed by club cells, which are unique to the lung, and in nonciliated columnar cells of the large and small bronchi and bronchioles (24, 32). CC16 reduces airway inflammation and oxidative stress and may also sequester some harmful substances deposited in the deep lung. Whereas most studies found that an acute lung injury increases CC16 in the serum (24, 32), others reported that serum CC16 can decrease it (29). Mechanisms underpinning serum CC16 variations are complex and depend on alveolar epithelial permeability alteration, club cell death, and changes in transcriptional activity within remaining club cells (24, 29, 32). In addition, the kinetics of CC16 variations are not well known and may depend on the lung irritant investigated (2, 24, 32). For instance, acute tobacco smoke exposure in a rat model has been shown to double serum CC16 within 2–4 h as a result of increased permeability of the lung-blood barrier (62). Thus, the serum CC16 measurements we obtained 30 min after exposure may have been followed by even larger rises thereafter. The transient increases in serum CC16 that we observed after vaping could have been due to direct lung leakage induced by increased epithelial permeability and/or decreased renal clearance (24). The latter possibility can be reasonably excluded, since serum creatinine was not modified by vaping or sham vaping in our participants. Because CC16 is secreted mainly within the respiratory tract, its presence in the vascular compartment and its observed increase as fast as 30 min after vaping can be explained only by assuming leakage from the lung into the bloodstream (24). The leakiness of lung proteins is mainly dependent on epithelial thickness and pore size (24). This could explain why serum SPD, which is nearly 40 times larger than CC16 (24), did not increase after acute vaping exposure. In addition to the osmotic and hygroscopic properties of e-cigarette aerosol (18, 19, 25), the low level of volatile carbonyls produced by thermal degradation of PG/GLY at high wattage could also injure airway epithelium and increase their permeability (25, 46, 58), resulting in subsequent intravascular leakage of CC16 (24, 32, 68). Thus, there are several reasons to believe that the increase in serum CC16 levels that we observed after vaping reflects epithelial dysfunction that might impair respiratory fluid regulation and pulmonary gas exchange (24, 28, 32, 68). This is also supported by the correlation we found between serum CC16 increase and Tcpo_2_ decrease after vaping without nicotine, suggesting that similar mechanisms induced epithelial dysfunction and pulmonary gas exchange disturbances in our participants.

### Pulmonary Gas Exchange and Vaping

Vaping with and without nicotine induced sustained decrements in Tcpo_2_, an indirect measurement of Po_2_ that depends on SkBF (65). However, SkBF was not significantly affected by vaping in our healthy participants, whereas their forced expiratory flows decreased. We also showed that in heavy smokers decreases in Tcpo_2_ seen after vaping was accompanied by concomitant decreases in Po_2_ and in oxygenated hemoglobin fraction. The latter changes strongly suggest acute perturbations in pulmonary gas exchange after vaping as a result of small airway constriction (12, 44). Development of ventilation-perfusion inequalities due to the underlying presence of areas with low ventilation-perfusion ratios is likely to account for the post-vaping decreases in Tcpo_2_ and Po_2_ (44). The effect of these low ventilation-perfusion units on Pco_2_ is classically less than the effect on Po_2_, because CO_2_ elimination is increased in the unit with a high ventilation-perfusion ratio (44, 66). This could explain why vaping did not increase Tcpco_2_ and Pco_2_ in healthy occasional smokers (first study) and in heavy smokers (second study), respectively. It should be noted that in the subgroup of heavy smokers where Tcpco_2_ data were available, the latter increased after nicotine-free vaping. The populations investigated in these two clinical studies were very different. In the first study, young and healthy occasional smokers were enrolled, whereas older heavy smokers suffering from CAD (65%) and COPD (30%) participated in the second study. In healthy humans, CO_2_ metabolism is finely balanced, and homeostasis is maintained through changes in breathing pattern (7, 66). In heavy smokers, the response of the respiratory center, muscles, and lungs could be less adequate than that of healthy subjects (7). This could explain why some older heavy smokers increased Tcpco_2_ and Pco_2_ after vaping without nicotine, while none of the young healthy occasional tobacco smokers did do so.

Several studies found that tobacco cigarette smoking also induces pulmonary gas exchange disturbances with similar changes in transcutaneous and arterial gas tensions to those we observed after vaping (26, 50, 51). Cigarette effects seem mediated mainly through the vasoconstrictive and hypoxic effect of carbon monoxide and toxic carbonyls produced by tobacco combustion (51). In contrast, e-cigarette vaping in real-life settings, even at high wattage, does not emit carbon monoxide and produces low levels of carbonyls (4, 16), which are unlikely the main contributors to the lung inflammation and gas exchange disturbances that we observed. There are several reasons to believe that a large amount of hyperosmolar and hygroscopic PG/GLY aerosol released during high wattage vaping can induce inflammation and alter the rheological properties of mucus/surfactant (11, 18, 19, 25, 37, 44, 45, 47, 52) with subsequent acute transient perturbations in pulmonary gas exchange comparable to those induced by nebulized hypertonic saline and mannitol challenge tests (31, 38).

### PG/GLY or Nicotine: Which Is the Culprit?

Vaping PG/GLY vehicles without nicotine caused decreases in Tcpo_2_ and lung inflammation. Vaping the same vehicles with nicotine increased the Tcpo_2_ decrement only slightly but induced a sustained Tcpco_2_ decrease after exposure, as a potential increase of minute ventilation induced by nicotine (23). Nicotine vaping had also shortened the duration of the rise in serum CC16 levels. The nicotinic activation of the cholinergic anti-inflammatory pathway at the level of the club cells could decrease the secretion of CC16 (20). This could also explain why in case of nicotine vaping there was no correlation between serum CC16 increase and Tcpo_2_ decrease in contrast to what was observed during nicotine-free vaping. In addition, nine participants in the nicotine vaping group complained of some degree of throat irritation. As an upper airway irritant (33), nicotine may decrease the depth of e-cigarette aerosol inhalation and thereby decrease its toxicity (14). In the same way, vaping with nicotine did not increase serum PG, in contrast to vaping without nicotine, suggesting that nicotine could modify the pattern of inhalation and thus the place of deposit in the lung (14), with subsequent change in kinetics absorption (30). Finally, it should be mentioned that the total nicotine intake after vaping in our first study was relatively high compared with those of other studies (53, 54, 55, 56, 57). Despite this high nicotine intake, the effects of vaping on serum CC16 and Tcpo_2_ appear largely attributable to the e-cigarette vehicles PG and GLY rather than to nicotine.

### Potential Effects of Chronic Vaping Exposure

Because of the newness of vaping and the multitude of devices, flavorings, and nicotine concentrations on the market, there is a lack of data regarding its long-term effects on the respiratory system (39). Fortunately, the acute changes in Tcpo_2_ and Po_2_ seen in these studies would not markedly affect arterial O_2_ content, as a Po_2_ of 60 mmHg provides ~90% hemoglobin saturation (12, 44). However, this effect could be more pronounced in patients with severe lung disease or might prove more deleterious with chronic vaping (21, 64). Deep lung hyperosmolar and hygroscopic PG/GLY deposit, oxidant chemicals, particulates, or flavorings can trigger lung inflammation (11, 18, 19, 25), with subsequent bronchoconstriction (25, 38) and airway remodeling (39). In case of chronic use, the latter phenomena could ease lower respiratory tract infection (36, 39) and promote asthma and COPD, as it is described with tobacco smoking or environmental pollution (11, 43). Thus, e-cigarette aerosol constituents could injure the respiratory system or worsen preexisting lung disease through a variety of mechanisms (11, 39). Although it is encouraging that few serious adverse events have been reported related to e-cigarette use during the years the products have been available (39), the potential e-cigarette health effects have to be carefully monitored for the coming years.

### Limitations

The main limitations of these two randomized, sham-controlled studies are the small number of subjects investigated as well as the lack of blinding during experimental exposures. The latter is an intrinsic limitation of any study where an aerosol is inhaled. In the main study, we assessed the effects of e-cigarettes in a cohort of healthy young participants. Occasional smokers with low cumulative cigarette pack-year smoking histories were enrolled instead of participants who have never smoked, because we believed the participants who have never smoked would not be able to vape e-cigarettes as required in this protocol. Thus, our results may not necessarily apply to other clinical situations. Nevertheless, we found very similar results in heavy smoker patients with substantial smoking histories. Although a crossover design was considered for the study investigating heavy smokers, it was discarded for ethical reasons (i.e., risk of arterial catheter thrombosis and radial artery vasospasm if the sessions were extended) (22). The healthy participants were carefully selected, and only those able to vape at high wattage were recruited; the majority of participants reported inhaling vapor directly into the lungs, without retaining it in the mouth (direct-to-lung inhalation). This practice is not common to all users of e-cigarettes. Although the majority of sub-ohm high-wattage vapers directly inhale PG/GLY aerosols (direct-to-lung inhalation), our clinical findings should not be extrapolated to more cautious users (58). Finally, to ensure a consistent exposure across subjects, only one type of e-cigarette device was used, which may not represent all high-output wattage devices. Flavorings were not administered in our studies, and their specific effects will require further investigation. But our findings are applicable to the vast majority of e-liquids that are composed of the PG/GLY vehicles (15).

### Conclusions

In conclusion, acute vaping of PG/GLY aerosols with and without nicotine at high wattage and in large amounts induces a sustained decrement in Tcpo_2_ and airway epithelial injury in young occasional tobacco smokers. The latter effect seems driven primarily by PG/GLY rather than by nicotine. These intense vaping conditions also elicit a decrease in Po_2_ in heavy smokers. Further studies are needed to confirm these observations in dedicated vapers to shed light on the precise pathogenetic mechanisms involved, and to identify the potential long-term consequences of e-cigarette usage.

## GRANTS

This study was supported by the “Fonds Erasme pour la Recherche Médicale,” Belgium (to M. Chaumont), the “Fondation pour la Chirurgie Cardiaque,” Belgium (to M. Chaumont), the “Fondation Emile Saucez-René Van Poucke,” Belgium (to M. Chaumont), the “Prix Docteur & Mrs Rene Tagnon,” Belgium (to M. Chaumont), the “Fondation IRIS,” Belgium (to M. Chaumont), the “Prix de l'Association André Vésale,” Belgium (to M. Chaumont), the “Fondation Drieghe-Miller,” Belgium (to M. Chaumont), a research grant from Astra Zeneca, Belgium (to P. van de Borne), the “Fonds Fruit de Deux Vies,” Belgium (to P. van de Borne), and the “Fond David and Alice Van Buuren,” Belgium (to P. van de Borne).

## DISCLOSURES

No conflicts of interest, financial or otherwise, are declared by the authors.

## AUTHOR CONTRIBUTIONS

M.C., P.v.d.B., W.Z., and N.D. conceived and designed research; M.C., A.B., G.D., R.B., Q.d.H., and W.Z. performed experiments; A.V.M., J.U., and E.S. analyzed data; M.C. and W.Z. interpreted results of experiments; M.C. prepared figures; M.C. drafted manuscript; M.C., P.v.d.B., A.B., and N.D. edited and revised manuscript; M.C., P.v.d.B., A.B., A.V.M., G.D., J.U., E.S., R.B., Q.d.H., W.Z., and N.D. approved final version of manuscript.
